# Telehealth and access to urgent care: maximising the benefits for patients with long-term conditions in England

**Published:** 2012-06-15

**Authors:** Hadleigh Stollar

**Affiliations:** NHS Direct, UK

**Keywords:** telehealth, telecoaching, health outcomes, empower

## Abstract

NHS Direct strives to provide a range of technology enabled interventions to support and empower patients across England. NHS Direct is able to offer a range of Telehealth services, which aim to enhance the health and well-being of patients with long-term conditions (LTC) and positively impact on the wider health economy. Our innovative LTC services, which have been developed alongside existing successful projects, provide patients with information about their condition, offering and informing choices whilst collaborating with local healthcare teams. These projects have been highly successful and show our ability to deliver at a large-scale.

- In South East Essex we provide a monitoring service for patients with COPD who are linked to equipment that monitors vital signs. The monitoring enables very early identification of possible changes to health state and through collaboration with local clinicians ensures actions are taken to prevent a deterioration that may in the past have led to a high cost hospital admission.
- In Leeds and Hull we provide a similar service with the addition of mental health monitoring. Many patients with LTCs also suffer high levels of anxiety and/or depression that can be as disabling as the physical health condition. Mental health is monitored through home-based technology and any early signs of deterioration are identified and managed before any serious harm occurs.
- In Nottingham we deliver ‘Nottingham Healthy Change’, which connects people who have cardiovascular disease risk factors to locally delivered lifestyle groups. Following an initial remote assessment, patients are referred to lifestyle change providers whilst taking part in a motivational coaching programme. This identifies the right lifestyle group(s) and ensures maximum benefit is achieved resulting in a healthier lifestyle and reduction is CVD risk factors. The scheme has been going just short of 12 months and already the PCT has asked to extend it.
- In Nottingham and Birmingham we provide telephone-based care management through nursing staff which seeks to support the local multi-disciplinary team by offering telephone based advice, motivation and clinical information.

Outcomes from existing NHS Direct services (which are general) show:

- Eighty-five percent of patients agreed or strongly agreed that the Telehealth service had helped them understand their condition.
- Eighty-eight percent of patients agreed or strongly agreed that it had helped them cope with their symptoms.
- Eighty-one percent of patients agreed or strongly agreed that the Telehealth service had helped to reduce anxiety.

In all these services there is evidence of reduced demand on expensive hospital and community services, improved quality of life for the patient and improved health outcomes. Lessons learnt have been invaluable in the development of new Telehealth services. In addition, NHS Directs involvement in the 111 pilots and other 111 services is identifying where Telehealth can become fully integrated ensuring seamless care for patients with LTCs both in and out of hours. In the future we are looking at other care pathways that would benefit from technology enabled support—from home delivered chemotherapy to supported discharge following surgery.

